# Supplementary material for: Assessment of the effectiveness of host depletion techniques for profiling fish skin microbiomes and metagenomic analysis
Source: Microbiol Spectr. 2025 Dec 22;14(2):e01838-25. doi: 10.1128/spectrum.01838-25 (PMC12889060; doi:10.1128/spectrum.01838-25)
Supplement: Supplemental material — Figures S1 to S4; Tables S1 to S3. [file spectrum.01838-25-s0001.docx]

# Supplementary Documents


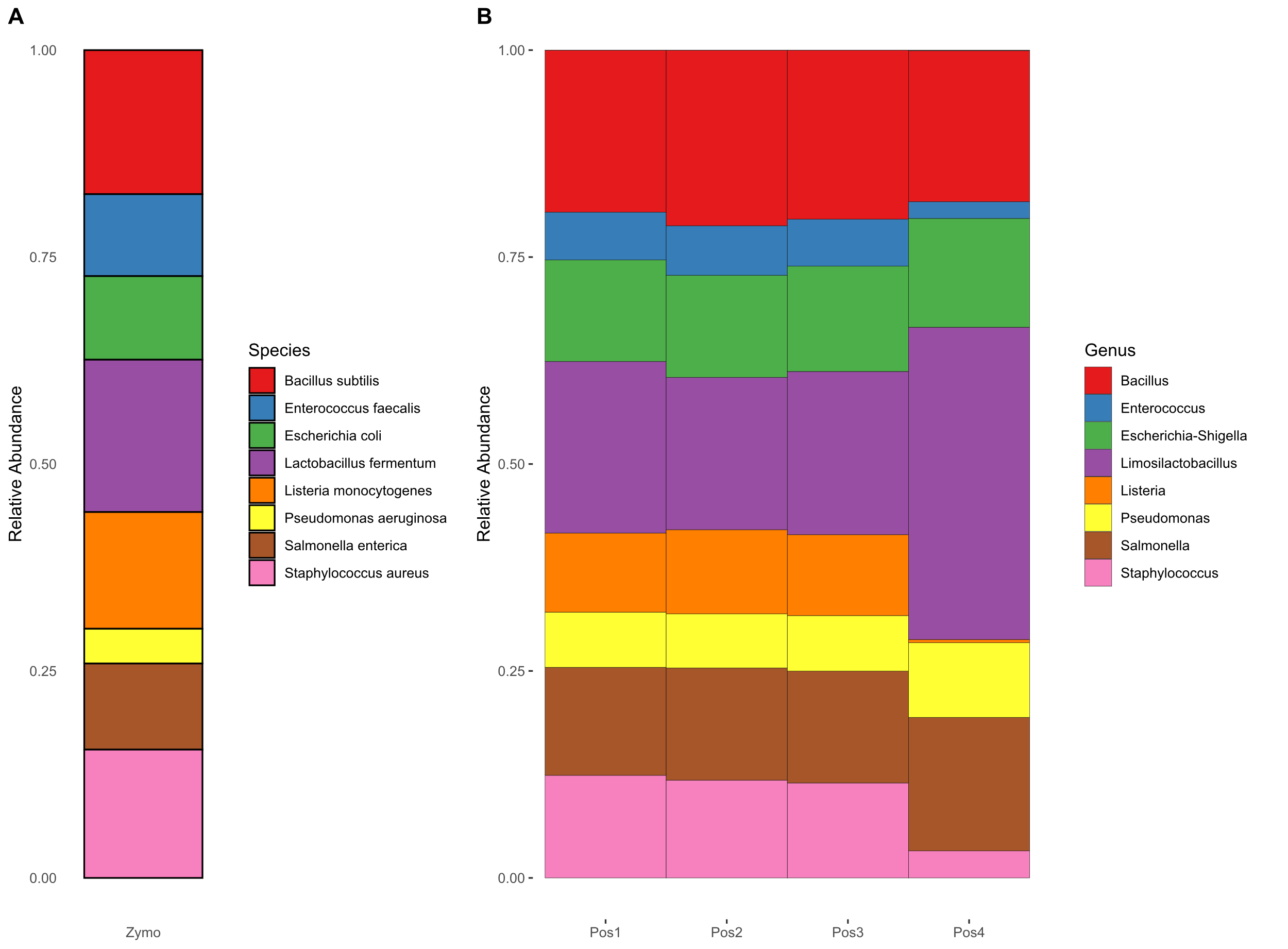


Supplementary Figure 1 Relative abundance of taxa in a ZymoBIOMICS Microbial Community Standard. A) Expected relative abundance B) DNA extraction of ZymoBIOMICS Microbial Community Standard as Positive controls. Taxa are only identified to a genus level.


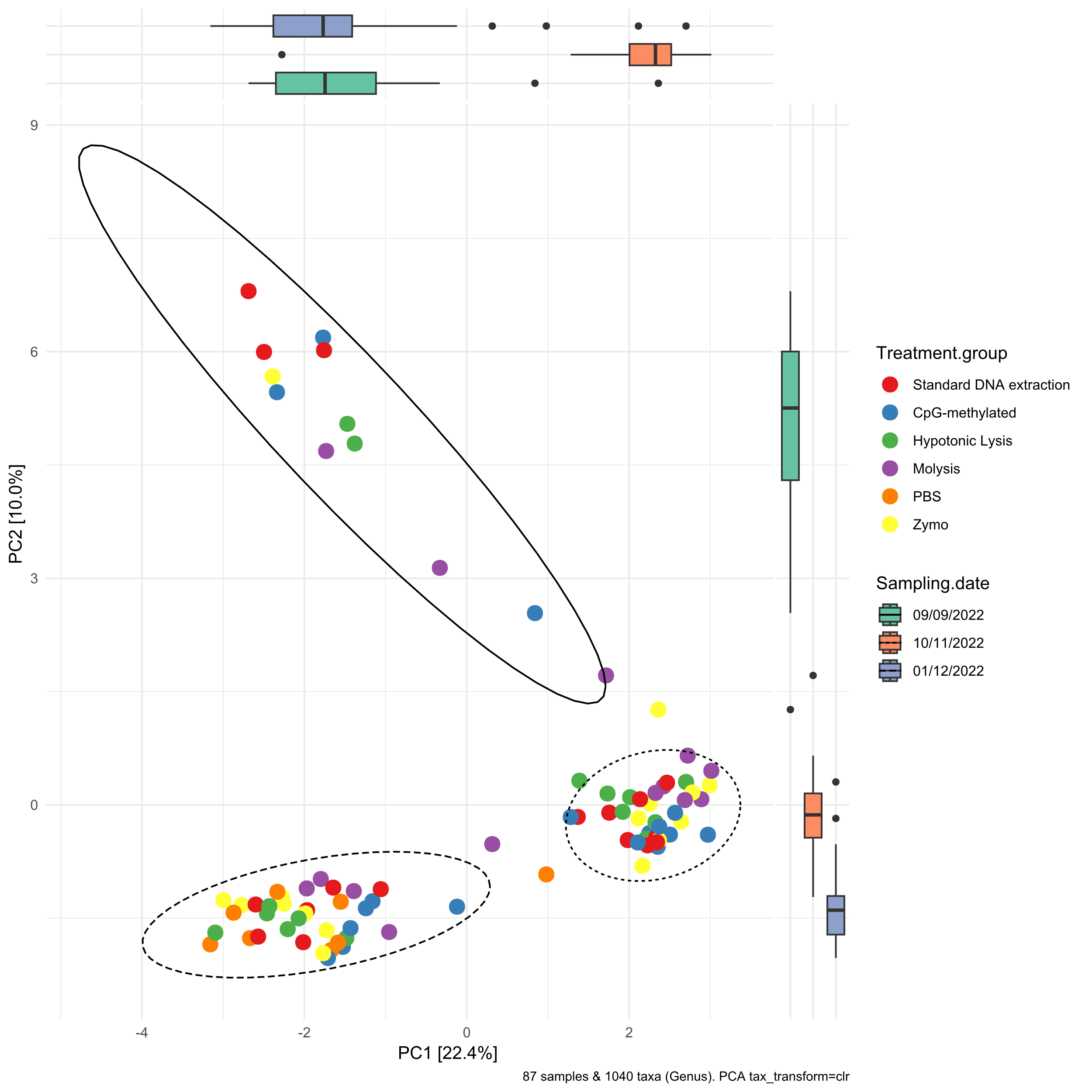


Supplementary Figure 2 PCA biplot of centre log-ratio transformed ASVs counts grouped by sampling date and treatment group.


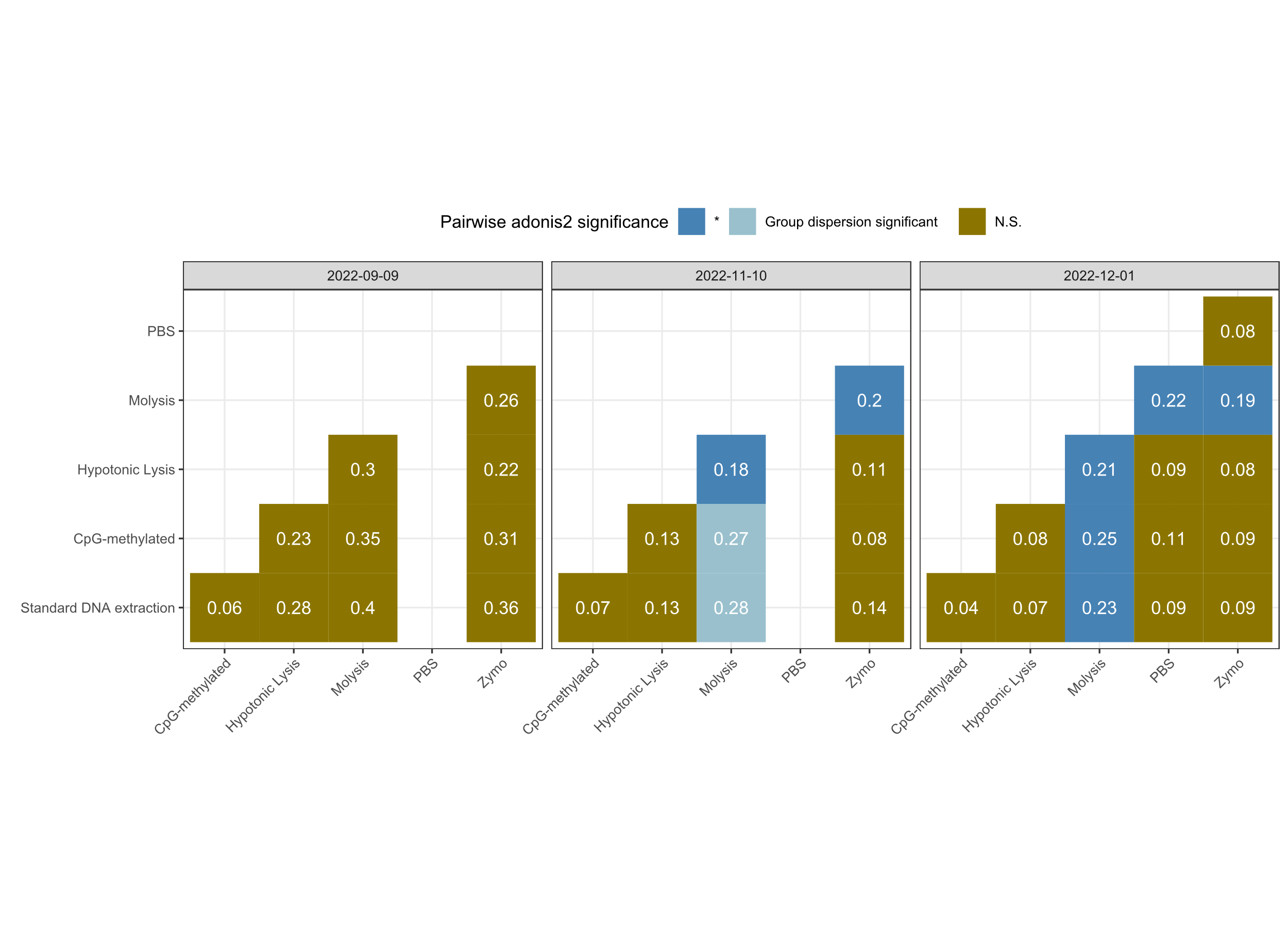


Supplementary Figure 3 Pairwise.adonis2 comparing beta-diversity of different host depletion treatment groups against each other from a Bray-Curtis dissimilarity matrix on compositional data aggregated at a genus level. Colours indicate significance of pairwise.adonis2 with blue as significant, light blue indicating pairwise adonis2 and test for group dispersion was significant so statistical test may be measuring variance rather than distance between groups. Dark brown indicates non-significant pairwise.adonis2, numbers representing R^2^ value indicating how well the model fitted the data.


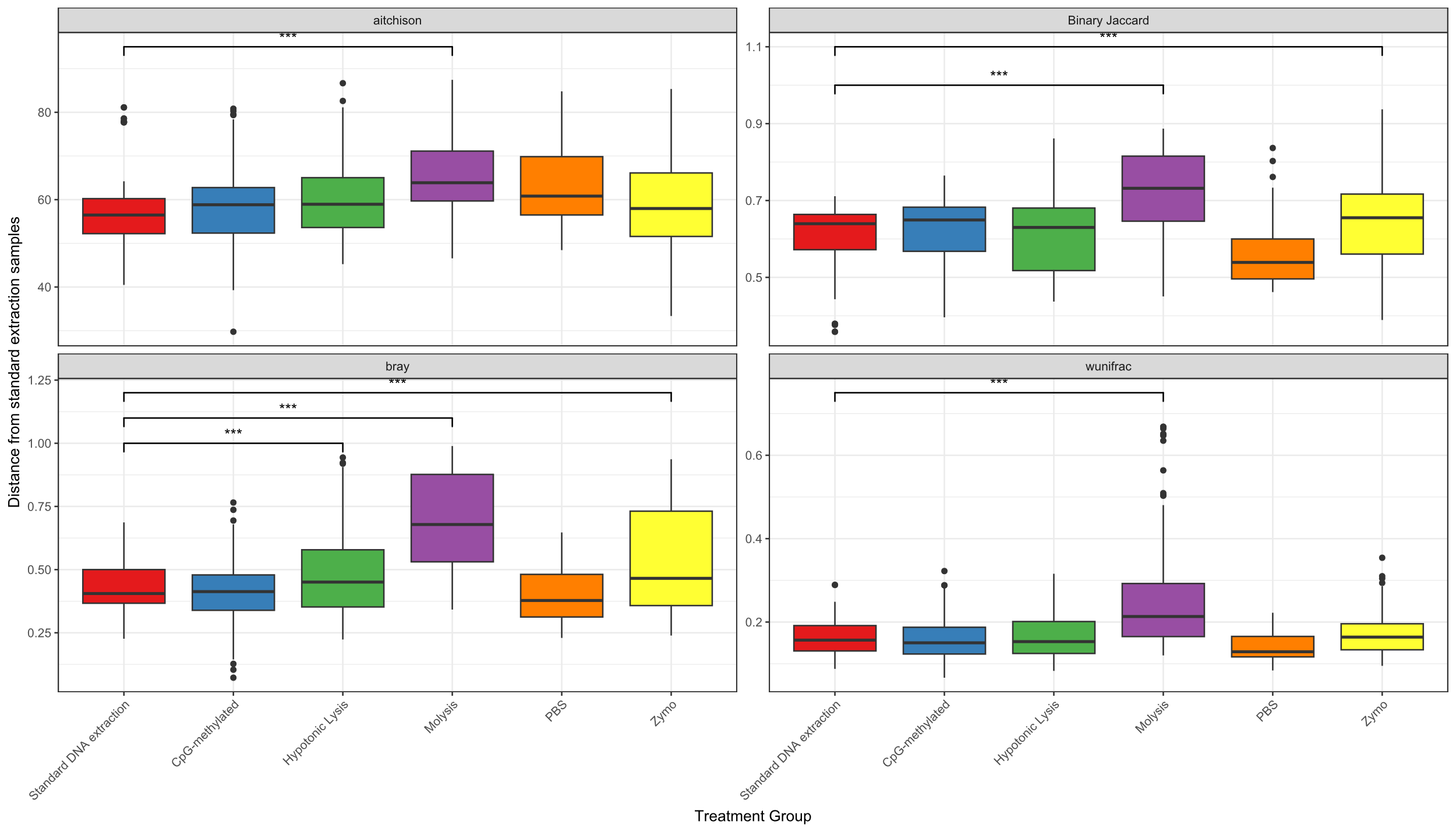


Supplementary Figure 4 The distance between the standard DNA extraction and host depletion treatment groups using Aitchison’s distance, Binary Jaccard, Bray-Curtis and Weighted UniFrac dissimilarity matrices. Significance is tested for each host depletion technique versus the standard DNA extraction control using a linear model corrected for multiple hypothesis testing. ***P ≤ 0.001.

Supplementary Table 1 16S amplicon forward and reverse primer sequences and combinations used within this study.

| Well Position | Forward Read Name | Forward Read Sequence | Reverse Read Name | Reverse Read Sequences |
| --- | --- | --- | --- | --- |
| A1 | 515fcbc61 | GTGGAGTCTCATGTGTGYCAGCMGCCGCGGTAA | 806rcbc147 | ACAACACTCCGACCGGACTACNVGGGTWTCTAAT |
| A2 | 515fcbc160 | AGTGTTTCGGACGTGTGYCAGCMGCCGCGGTAA | 806rcbc220 | GTCGTCCAAATGCCGGACTACNVGGGTWTCTAAT |
| A3 | 515fcbc151 | AGCTATGTATGGGTGTGYCAGCMGCCGCGGTAA | 806rcbc202 | GTCTTCAGCAAGCCGGACTACNVGGGTWTCTAAT |
| A4 | 515fcbc38 | ACCATAGCTCCGGTGTGYCAGCMGCCGCGGTAA | 806rcbc247 | AGGTCCAAATCACCGGACTACNVGGGTWTCTAAT |
| A5 | 515fcbc69 | TATCGACACAAGGTGTGYCAGCMGCCGCGGTAA | 806rcbc132 | TCCTCGAGCGATCCGGACTACNVGGGTWTCTAAT |
| A6 | 515fcbc152 | ACGGGTCATCATGTGTGYCAGCMGCCGCGGTAA | 806rcbc124 | CCTGCGAAGTATCCGGACTACNVGGGTWTCTAAT |
| A7 | 515fcbc52 | ACTCACAGGAATGTGTGYCAGCMGCCGCGGTAA | 806rcbc244 | GAGACGTGTTCTCCGGACTACNVGGGTWTCTAAT |
| A8 | 515fcbc34 | TTGCGTTAGCAGGTGTGYCAGCMGCCGCGGTAA | 806rcbc115 | TACCGAAGGTATCCGGACTACNVGGGTWTCTAAT |
| A9 | 515fcbc142 | GCATAGCATCAAGTGTGYCAGCMGCCGCGGTAA | 806rcbc4 | TGGTCAACGATACCGGACTACNVGGGTWTCTAAT |
| A10 | 515fcbc146 | AGAGTCTTGCCAGTGTGYCAGCMGCCGCGGTAA | 806rcbc167 | CGCTACAACTCGCCGGACTACNVGGGTWTCTAAT |
| A11 | 515fcbc23 | GCAACACCATCCGTGTGYCAGCMGCCGCGGTAA | 806rcbc135 | AGCAACATTGCACCGGACTACNVGGGTWTCTAAT |
| A12 | 515fcbc138 | GTAGAGGTAGAGGTGTGYCAGCMGCCGCGGTAA | 806rcbc153 | GAAACATCCCACCCGGACTACNVGGGTWTCTAAT |
| B1 | 515fcbc145 | TGAACCCTATGGGTGTGYCAGCMGCCGCGGTAA | 806rcbc111 | AGCTGTCAAGCTCCGGACTACNVGGGTWTCTAAT |
| B2 | 515fcbc15 | GAATACCAAGTCGTGTGYCAGCMGCCGCGGTAA | 806rcbc146 | AGAGTCTTGCCACCGGACTACNVGGGTWTCTAAT |
| B3 | 515fcbc66 | CTCACCTAGGAAGTGTGYCAGCMGCCGCGGTAA | 806rcbc83 | GTTCTCTTCTCGCCGGACTACNVGGGTWTCTAAT |
| B4 | 515fcbc125 | TTCTCTCGACATGTGTGYCAGCMGCCGCGGTAA | 806rcbc246 | TATGCCAGAGATCCGGACTACNVGGGTWTCTAAT |
| B5 | 515fcbc154 | CGTACTCTCGAGGTGTGYCAGCMGCCGCGGTAA | 806rcbc66 | CTCACCTAGGAACCGGACTACNVGGGTWTCTAAT |
| B6 | 515fcbc150 | ACGCGAACTAATGTGTGYCAGCMGCCGCGGTAA | 806rcbc49 | GGCCAGTTCCTACCGGACTACNVGGGTWTCTAAT |
| B7 | 515fcbc100 | GATGTATGTGGTGTGTGYCAGCMGCCGCGGTAA | 806rcbc125 | TTCTCTCGACATCCGGACTACNVGGGTWTCTAAT |
| B8 | 515fcbc30 | AGATTGACCAACGTGTGYCAGCMGCCGCGGTAA | 806rcbc270 | GATCTCTGGGTACCGGACTACNVGGGTWTCTAAT |
| B9 | 515fcbc62 | GCTCGAAGATTCGTGTGYCAGCMGCCGCGGTAA | 806rcbc35 | TACGAGCCCTAACCGGACTACNVGGGTWTCTAAT |
| B10 | 515fcbc113 | TACTCGGGAACTGTGTGYCAGCMGCCGCGGTAA | 806rcbc186 | CTGATCCATCTTCCGGACTACNVGGGTWTCTAAT |
| B11 | 515fcbc123 | ATGTGTGTAGACGTGTGYCAGCMGCCGCGGTAA | 806rcbc221 | CAACGTGCTCCACCGGACTACNVGGGTWTCTAAT |
| B12 | 515fcbc97 | TGTGCGATAACAGTGTGYCAGCMGCCGCGGTAA | 806rcbc173 | AACGTAGGCTCTCCGGACTACNVGGGTWTCTAAT |
| C1 | 515fcbc99 | GCCTAGCCCAATGTGTGYCAGCMGCCGCGGTAA | 806rcbc86 | GTTGTTCTGGGACCGGACTACNVGGGTWTCTAAT |
| C2 | 515fcbc46 | TGTGAATTCGGAGTGTGYCAGCMGCCGCGGTAA | 806rcbc8 | ATCCTTTGGTTCCCGGACTACNVGGGTWTCTAAT |
| C3 | 515fcbc19 | CCAATACGCCTGGTGTGYCAGCMGCCGCGGTAA | 806rcbc31 | AGTTACGAGCTACCGGACTACNVGGGTWTCTAAT |
| C4 | 515fcbc158 | GATCACGAGAGGGTGTGYCAGCMGCCGCGGTAA | 806rcbc175 | TCGTCAAACCCGCCGGACTACNVGGGTWTCTAAT |
| C5 | 515fcbc103 | GCGAGCGAAGTAGTGTGYCAGCMGCCGCGGTAA | 806rcbc108 | GCACACCTGATACCGGACTACNVGGGTWTCTAAT |
| C6 | 515fcbc6 | GTCGTGTAGCCTGTGTGYCAGCMGCCGCGGTAA | 806rcbc6 | GTCGTGTAGCCTCCGGACTACNVGGGTWTCTAAT |
| C7 | 515fcbc112 | GAGAGCAACAGAGTGTGYCAGCMGCCGCGGTAA | 806rcbc87 | GGACTTCCAGCTCCGGACTACNVGGGTWTCTAAT |
| C8 | 515fcbc63 | AGGCTTACGTGTGTGTGYCAGCMGCCGCGGTAA | 806rcbc89 | CTGCTATTCCTCCCGGACTACNVGGGTWTCTAAT |
| C9 | 515fcbc17 | TAACGTGTGTGCGTGTGYCAGCMGCCGCGGTAA | 806rcbc26 | AGTCGTGCACATCCGGACTACNVGGGTWTCTAAT |
| C10 | 515fcbc106 | TCTTGGAGGTCAGTGTGYCAGCMGCCGCGGTAA | 806rcbc219 | TGGTTGGTTACGCCGGACTACNVGGGTWTCTAAT |
| C11 | 515fcbc94 | TTGGCTCTATTCGTGTGYCAGCMGCCGCGGTAA | 806rcbc234 | CTCGTGAATGACCCGGACTACNVGGGTWTCTAAT |
| C12 | 515fcbc108 | GCACACCTGATAGTGTGYCAGCMGCCGCGGTAA | 806rcbc172 | CACTGGTGCATACCGGACTACNVGGGTWTCTAAT |
| D1 | 515fcbc117 | GTATTTCGGACGGTGTGYCAGCMGCCGCGGTAA | 806rcbc105 | ACTTGGTGTAAGCCGGACTACNVGGGTWTCTAAT |
| D2 | 515fcbc12 | TGCATACACTGGGTGTGYCAGCMGCCGCGGTAA | 806rcbc80 | TAGTATGCGCAACCGGACTACNVGGGTWTCTAAT |
| D3 | 515fcbc140 | GGTTATTTGGCGGTGTGYCAGCMGCCGCGGTAA | 806rcbc17 | TAACGTGTGTGCCCGGACTACNVGGGTWTCTAAT |
| D4 | 515fcbc80 | TAGTATGCGCAAGTGTGYCAGCMGCCGCGGTAA | 806rcbc144 | TTAGAGCCATGCCCGGACTACNVGGGTWTCTAAT |
| D5 | 515fcbc28 | CGAGGGAAAGTCGTGTGYCAGCMGCCGCGGTAA | 806rcbc16 | GTAGATCGTGTACCGGACTACNVGGGTWTCTAAT |
| D6 | 515fcbc137 | CAGAAATGTGTCGTGTGYCAGCMGCCGCGGTAA | 806rcbc237 | GGTTTAACACGCCCGGACTACNVGGGTWTCTAAT |
| D7 | 515fcbc8 | ATCCTTTGGTTCGTGTGYCAGCMGCCGCGGTAA | 806rcbc138 | GTAGAGGTAGAGCCGGACTACNVGGGTWTCTAAT |
| D8 | 515fcbc156 | TCGTGCGTGTTGGTGTGYCAGCMGCCGCGGTAA | 806rcbc11 | AATTGTGTCGGACCGGACTACNVGGGTWTCTAAT |
| D9 | 515fcbc10 | ACCGGTATGTACGTGTGYCAGCMGCCGCGGTAA | 806rcbc70 | GATTCCGGCTCACCGGACTACNVGGGTWTCTAAT |
| D10 | 515fcbc45 | TCGGAATTAGACGTGTGYCAGCMGCCGCGGTAA | 806rcbc5 | ATCGCACAGTAACCGGACTACNVGGGTWTCTAAT |
| D11 | 515fcbc96 | TACCGCTTCTTCGTGTGYCAGCMGCCGCGGTAA | 806rcbc185 | GGTTCGGTCCATCCGGACTACNVGGGTWTCTAAT |
| D12 | 515fcbc55 | TGTCGCAAATAGGTGTGYCAGCMGCCGCGGTAA | 806rcbc203 | CGGATAACCTCCCCGGACTACNVGGGTWTCTAAT |
| E1 | 515fcbc131 | ATCAGTACTAGGGTGTGYCAGCMGCCGCGGTAA | 806rcbc211 | TGCACGTGATAACCGGACTACNVGGGTWTCTAAT |
| E2 | 515fcbc7 | AGCGGAGGTTAGGTGTGYCAGCMGCCGCGGTAA | 806rcbc252 | CTATCATCCTCACCGGACTACNVGGGTWTCTAAT |
| E3 | 515fcbc89 | CTGCTATTCCTCGTGTGYCAGCMGCCGCGGTAA | 806rcbc197 | ATTCGGTAGTGCCCGGACTACNVGGGTWTCTAAT |
| E4 | 515fcbc134 | TGCAGCAAGATTGTGTGYCAGCMGCCGCGGTAA | 806rcbc206 | GCCTGTCTGCAACCGGACTACNVGGGTWTCTAAT |
| E5 | 515fcbc85 | GCGTTCTAGCTGGTGTGYCAGCMGCCGCGGTAA | 806rcbc263 | GATGCTGCCGTTCCGGACTACNVGGGTWTCTAAT |
| E6 | 515fcbc39 | TCGACATCTCTTGTGTGYCAGCMGCCGCGGTAA | 806rcbc41 | GAGCCATCTGTACCGGACTACNVGGGTWTCTAAT |
| E7 | 515fcbc11 | AATTGTGTCGGAGTGTGYCAGCMGCCGCGGTAA | 806rcbc248 | ACCGTGCTCACACCGGACTACNVGGGTWTCTAAT |
| E8 | 515fcbc25 | CGAGCAATCCTAGTGTGYCAGCMGCCGCGGTAA | 806rcbc235 | AGGTGAGTTCTACCGGACTACNVGGGTWTCTAAT |
| E9 | 515fcbc50 | GATGTTCGCTAGGTGTGYCAGCMGCCGCGGTAA | 806rcbc79 | GTGGTGGTTTCCCCGGACTACNVGGGTWTCTAAT |
| E10 | 515fcbc49 | GGCCAGTTCCTAGTGTGYCAGCMGCCGCGGTAA | 806rcbc78 | GTACGATATGACCCGGACTACNVGGGTWTCTAAT |
| E11 | 515fcbc20 | GATCTGCGATCCGTGTGYCAGCMGCCGCGGTAA | 806rcbc65 | ACTTCCAACTTCCCGGACTACNVGGGTWTCTAAT |
| E12 | 515fcbc13 | AGTCGAACGAGGGTGTGYCAGCMGCCGCGGTAA | 806rcbc54 | GTCGACAGAGGACCGGACTACNVGGGTWTCTAAT |
| F1 | 515fcbc130 | GCCAACAACCATGTGTGYCAGCMGCCGCGGTAA | 806rcbc258 | GAGAGTCCACTTCCGGACTACNVGGGTWTCTAAT |
| F2 | 515fcbc107 | TCACCTCCTTGTGTGTGYCAGCMGCCGCGGTAA | 806rcbc251 | CCTTGACCGATGCCGGACTACNVGGGTWTCTAAT |
| F3 | 515fcbc78 | GTACGATATGACGTGTGYCAGCMGCCGCGGTAA | 806rcbc157 | GTTATCGCATGGCCGGACTACNVGGGTWTCTAAT |
| F4 | 515fcbc120 | AGTAGCGGAAGAGTGTGYCAGCMGCCGCGGTAA | 806rcbc12 | TGCATACACTGGCCGGACTACNVGGGTWTCTAAT |
| F5 | 515fcbc35 | TACGAGCCCTAAGTGTGYCAGCMGCCGCGGTAA | 806rcbc141 | GGATCGTAATACCCGGACTACNVGGGTWTCTAAT |
| F6 | 515fcbc139 | CGTGATCCGCTAGTGTGYCAGCMGCCGCGGTAA | 806rcbc69 | TATCGACACAAGCCGGACTACNVGGGTWTCTAAT |
| F7 | 515fcbc102 | GTCACGGACATTGTGTGYCAGCMGCCGCGGTAA | 806rcbc261 | GGTCTCCTACAGCCGGACTACNVGGGTWTCTAAT |
| F8 | 515fcbc105 | ACTTGGTGTAAGGTGTGYCAGCMGCCGCGGTAA | 806rcbc145 | TGAACCCTATGGCCGGACTACNVGGGTWTCTAAT |
| F9 | 515fcbc40 | GAACACTTTGGAGTGTGYCAGCMGCCGCGGTAA | 806rcbc239 | GCCACGACTTACCCGGACTACNVGGGTWTCTAAT |
| F10 | 515fcbc48 | TACTACGTGGCCGTGTGYCAGCMGCCGCGGTAA | 806rcbc18 | CATTATGGCGTGCCGGACTACNVGGGTWTCTAAT |
| F11 | 515fcbc82 | ATGGCTGTCAGTGTGTGYCAGCMGCCGCGGTAA | 806rcbc44 | TAATACGGATCGCCGGACTACNVGGGTWTCTAAT |
| F12 | 515fcbc16 | GTAGATCGTGTAGTGTGYCAGCMGCCGCGGTAA | 806rcbc96 | TACCGCTTCTTCCCGGACTACNVGGGTWTCTAAT |
| G1 | 515fcbc27 | GTATCTGCGCGTGTGTGYCAGCMGCCGCGGTAA | 806rcbc22 | CAAACAACAGCTCCGGACTACNVGGGTWTCTAAT |
| G2 | 515fcbc136 | GATGTGGTGTTAGTGTGYCAGCMGCCGCGGTAA | 806rcbc61 | GTGGAGTCTCATCCGGACTACNVGGGTWTCTAAT |
| G3 | 515fcbc81 | TGCGCTGAATGTGTGTGYCAGCMGCCGCGGTAA | 806rcbc142 | GCATAGCATCAACCGGACTACNVGGGTWTCTAAT |
| G4 | 515fcbc65 | ACTTCCAACTTCGTGTGYCAGCMGCCGCGGTAA | 806rcbc207 | ACTGATGGCCTCCCGGACTACNVGGGTWTCTAAT |
| G5 | 515fcbc4 | TGGTCAACGATAGTGTGYCAGCMGCCGCGGTAA | 806rcbc257 | CCAGATATAGCACCGGACTACNVGGGTWTCTAAT |
| G6 | 515fcbc1 | ACGAGACTGATTGTGTGYCAGCMGCCGCGGTAA | 806rcbc205 | GACTTCATGCGACCGGACTACNVGGGTWTCTAAT |
| G7 | 515fcbc72 | GGTGACTAGTTCGTGTGYCAGCMGCCGCGGTAA | 806rcbc256 | CTTAGGCATGTGCCGGACTACNVGGGTWTCTAAT |
| G8 | 515fcbc70 | GATTCCGGCTCAGTGTGYCAGCMGCCGCGGTAA | 806rcbc67 | GTGTTGTCGTGCCCGGACTACNVGGGTWTCTAAT |
| G9 | 515fcbc87 | GGACTTCCAGCTGTGTGYCAGCMGCCGCGGTAA | 806rcbc30 | AGATTGACCAACCCGGACTACNVGGGTWTCTAAT |
| G10 | 515fcbc116 | CACTCATCATTCGTGTGYCAGCMGCCGCGGTAA | 806rcbc245 | TATCACCGGCACCCGGACTACNVGGGTWTCTAAT |
| G11 | 515fcbc157 | GTTATCGCATGGGTGTGYCAGCMGCCGCGGTAA | 806rcbc231 | GTGTTCCCAGAACCGGACTACNVGGGTWTCTAAT |
| G12 | 515fcbc84 | CGTAAGATGCCTGTGTGYCAGCMGCCGCGGTAA | 806rcbc201 | CGACTCTAAACGCCGGACTACNVGGGTWTCTAAT |
| H1 | 515fcbc155 | TCAGTTCTCGTTGTGTGYCAGCMGCCGCGGTAA | 806rcbc241 | GCCGTAAACTTGCCGGACTACNVGGGTWTCTAAT |
| H2 | 515fcbc143 | GTGTTAGATGTGGTGTGYCAGCMGCCGCGGTAA | 806rcbc25 | CGAGCAATCCTACCGGACTACNVGGGTWTCTAAT |
| H3 | 515fcbc51 | CTATCTCCTGTCGTGTGYCAGCMGCCGCGGTAA | 806rcbc101 | ACTCCTTGTGTTCCGGACTACNVGGGTWTCTAAT |
| H4 | 515fcbc149 | ACGACTGCATAAGTGTGYCAGCMGCCGCGGTAA | 806rcbc266 | TGGCTTTCTATCCCGGACTACNVGGGTWTCTAAT |
| H5 | 515fcbc18 | CATTATGGCGTGGTGTGYCAGCMGCCGCGGTAA | 806rcbc268 | GAGCGTATCCATCCGGACTACNVGGGTWTCTAAT |
| H6 | 515fcbc93 | TGGAGTAGGTGGGTGTGYCAGCMGCCGCGGTAA | 806rcbc151 | AGCTATGTATGGCCGGACTACNVGGGTWTCTAAT |
| H7 | 515fcbc144 | TTAGAGCCATGCGTGTGYCAGCMGCCGCGGTAA | 806rcbc260 | ACGTGTAGGCTTCCGGACTACNVGGGTWTCTAAT |
| H8 | 515fcbc133 | ACCCAAGCGTTAGTGTGYCAGCMGCCGCGGTAA | 806rcbc154 | CGTACTCTCGAGCCGGACTACNVGGGTWTCTAAT |
| H9 | 515fcbc101 | ACTCCTTGTGTTGTGTGYCAGCMGCCGCGGTAA | 806rcbc85 | GCGTTCTAGCTGCCGGACTACNVGGGTWTCTAAT |
| H10 | 515fcbc29 | CAAATTCGGGATGTGTGYCAGCMGCCGCGGTAA | 806rcbc23 | GCAACACCATCCCCGGACTACNVGGGTWTCTAAT |
| H11 | 515fcbc83 | GTTCTCTTCTCGGTGTGYCAGCMGCCGCGGTAA | 806rcbc149 | ACGACTGCATAACCGGACTACNVGGGTWTCTAAT |
| H12 | 515fcbc115 | TACCGAAGGTATGTGTGYCAGCMGCCGCGGTAA | 806rcbc130 | GCCAACAACCATCCGGACTACNVGGGTWTCTAAT |

Supplementary Table 2 Pairwise.adonis2 comparing beta-diversity of different host depletion treatment groups against each other from a Bray-Curtis dissimilarity matrix on compositional data aggregated at a genus level. Beta dispersion statistics indicating test for group dispersion was significant so statistical test may be measuring variance rather than distance between groups (bdisp_sig). *** P < 0.001, ** P < 0.01, * P < 0.05; N.S. Not significant (P > 0.05).

| Comparison | | P adjusted beta dispersion | Sampling date | Beta dispersion significant (p < 0.05) | R2 | P adjusted pairwise.adonis2 | Significance pairwise.adonis2 |
| --- | --- | --- | --- | --- | --- | --- | --- |
| CpG-methylated | Standard DNA extraction | 0.058 | 09/09/2022 | N.S. | 0.06 | 0.742 | N.S. |
| Hypotonic Lysis | CpG-methylated | 0.025 | 09/09/2022 | * | 0.23 | 0.721 | bdisp_sig |
| Hypotonic Lysis | Standard DNA extraction | 0.001 | 09/09/2022 | *** | 0.28 | 0.560 | bdisp_sig |
| Molysis | CpG-methylated | 0.003 | 09/09/2022 | ** | 0.35 | 0.233 | bdisp_sig |
| Molysis | Hypotonic Lysis | 0.392 | 09/09/2022 | N.S. | 0.30 | 0.729 | N.S. |
| Molysis | Standard DNA extraction | <0.001 | 09/09/2022 | *** | 0.40 | 0.233 | bdisp_sig |
| Zymo | CpG-methylated | <0.001 | 09/09/2022 | *** | 0.31 | 0.368 | bdisp_sig |
| Zymo | Hypotonic Lysis | <0.001 | 09/09/2022 | *** | 0.22 | 1.000 | bdisp_sig |
| Zymo | Molysis | <0.001 | 09/09/2022 | *** | 0.26 | 0.729 | bdisp_sig |
| Zymo | Standard DNA extraction | <0.001 | 09/09/2022 | *** | 0.36 | 0.233 | bdisp_sig |
| CpG-methylated | Standard DNA extraction | 1.000 | 10/11/2022 | N.S. | 0.07 | 0.642 | N.S. |
| Hypotonic Lysis | CpG-methylated | 0.710 | 10/11/2022 | N.S. | 0.13 | 0.200 | N.S. |
| Hypotonic Lysis | Standard DNA extraction | 0.693 | 10/11/2022 | N.S. | 0.13 | 0.201 | N.S. |
| Molysis | CpG-methylated | 0.003 | 10/11/2022 | ** | 0.27 | 0.012 | bdisp_sig |
| Molysis | Hypotonic Lysis | 0.104 | 10/11/2022 | N.S. | 0.18 | 0.031 | * |
| Molysis | Standard DNA extraction | 0.002 | 10/11/2022 | ** | 0.28 | 0.012 | bdisp_sig |
| Zymo | CpG-methylated | 0.195 | 10/11/2022 | N.S. | 0.08 | 0.456 | N.S. |
| Zymo | Hypotonic Lysis | 0.916 | 10/11/2022 | N.S. | 0.11 | 0.368 | N.S. |
| Zymo | Molysis | 0.400 | 10/11/2022 | N.S. | 0.20 | 0.020 | * |
| Zymo | Standard DNA extraction | 0.185 | 10/11/2022 | N.S. | 0.14 | 0.066 | N.S. |
| CpG-methylated | Standard DNA extraction | 1.000 | 01/12/2022 | N.S. | 0.04 | 0.961 | N.S. |
| Hypotonic Lysis | CpG-methylated | 0.950 | 01/12/2022 | N.S. | 0.08 | 0.689 | N.S. |
| Hypotonic Lysis | Standard DNA extraction | 0.955 | 01/12/2022 | N.S. | 0.07 | 0.721 | N.S. |
| Molysis | CpG-methylated | 0.973 | 01/12/2022 | N.S. | 0.25 | 0.020 | * |
| Molysis | Hypotonic Lysis | 1.000 | 01/12/2022 | N.S. | 0.21 | 0.018 | * |
| Molysis | Standard DNA extraction | 0.976 | 01/12/2022 | N.S. | 0.23 | 0.035 | * |
| PBS | CpG-methylated | 1.000 | 01/12/2022 | N.S. | 0.11 | 0.249 | N.S. |
| PBS | Hypotonic Lysis | 0.923 | 01/12/2022 | N.S. | 0.09 | 0.264 | N.S. |
| PBS | Molysis | 0.960 | 01/12/2022 | N.S. | 0.22 | 0.012 | * |
| PBS | Standard DNA extraction | 1.000 | 01/12/2022 | N.S. | 0.09 | 0.447 | N.S. |
| Zymo | CpG-methylated | 1.000 | 01/12/2022 | N.S. | 0.09 | 0.447 | N.S. |
| Zymo | Hypotonic Lysis | 0.990 | 01/12/2022 | N.S. | 0.08 | 0.591 | N.S. |
| Zymo | Molysis | 0.996 | 01/12/2022 | N.S. | 0.19 | 0.020 | * |
| Zymo | PBS | 0.999 | 01/12/2022 | N.S. | 0.08 | 0.447 | N.S. |
| Zymo | Standard DNA extraction | 1.000 | 01/12/2022 | N.S. | 0.09 | 0.447 | N.S. |

Supplementary Table 3 Differential abundance of bacterial phyla, order and class present in host depleted fish skin microbiome treatment groups at least 1% total abundance determined by a linear model corrected for multiple hypothesis testing compared to untreated fish skin microbiome DNA extractions. Formula “taxa~ Treatment.group + (1 | Sampling.date)”

| **term** | **taxon** | **rank** | **formula** | **effect** | **group** | **estimate** | **std.error** | **statistic** | **df** | **p.value** | **p.adj** |
| --- | --- | --- | --- | --- | --- | --- | --- | --- | --- | --- | --- |
| Treatment.groupCpG-methylated | P: Proteobacteria | Phylum | `P: Proteobacteria`~Treatment.group + (1 \| Sampling.date) | fixed | *NA* | 1214.353 | 10717.077 | 0.113 | 79.314 | 0.91 | 0.92 |
| Treatment.groupHypotonic Lysis | P: Proteobacteria | Phylum | `P: Proteobacteria`~Treatment.group + (1 \| Sampling.date) | fixed | *NA* | 5056.91 | 11075.998 | 0.457 | 79.895 | 0.649 | 0.812 |
| Treatment.groupMolysis | P: Proteobacteria | Phylum | `P: Proteobacteria`~Treatment.group + (1 \| Sampling.date) | fixed | *NA* | -9246.437 | 11277.425 | -0.82 | 79.39 | 0.415 | 0.754 |
| Treatment.groupPBS | P: Proteobacteria | Phylum | `P: Proteobacteria`~Treatment.group + (1 \| Sampling.date) | fixed | *NA* | 9369.266 | 13600.938 | 0.689 | 66.311 | 0.493 | 0.772 |
| Treatment.groupZymo | P: Proteobacteria | Phylum | `P: Proteobacteria`~Treatment.group + (1 \| Sampling.date) | fixed | *NA* | 22801.464 | 10887.582 | 2.094 | 79.686 | 0.039 | 0.328 |
| sd__(Intercept) | P: Proteobacteria | Phylum | `P: Proteobacteria`~Treatment.group + (1 \| Sampling.date) | ran_pars | Sampling.date | 3341.383 | *NA* | *NA* | *NA* | *NA* | *NA* |
| sd__Observation | P: Proteobacteria | Phylum | `P: Proteobacteria`~Treatment.group + (1 \| Sampling.date) | ran_pars | Residual | 31245.379 | *NA* | *NA* | *NA* | *NA* | *NA* |
| Treatment.groupCpG-methylated | P: Firmicutes | Phylum | `P: Firmicutes`~Treatment.group + (1 \| Sampling.date) | fixed | *NA* | -2898.353 | 4716.883 | -0.614 | 79.09 | 0.541 | 0.772 |
| Treatment.groupHypotonic Lysis | P: Firmicutes | Phylum | `P: Firmicutes`~Treatment.group + (1 \| Sampling.date) | fixed | *NA* | -8527.955 | 4884.053 | -1.746 | 79.178 | 0.085 | 0.423 |
| Treatment.groupMolysis | P: Firmicutes | Phylum | `P: Firmicutes`~Treatment.group + (1 \| Sampling.date) | fixed | *NA* | -7287.625 | 4965.071 | -1.468 | 79.11 | 0.146 | 0.529 |
| Treatment.groupPBS | P: Firmicutes | Phylum | `P: Firmicutes`~Treatment.group + (1 \| Sampling.date) | fixed | *NA* | -6382.7 | 6214.316 | -1.027 | 80.355 | 0.307 | 0.668 |
| Treatment.groupZymo | P: Firmicutes | Phylum | `P: Firmicutes`~Treatment.group + (1 \| Sampling.date) | fixed | *NA* | -9588.197 | 4798.402 | -1.998 | 79.164 | 0.049 | 0.328 |
| sd__(Intercept) | P: Firmicutes | Phylum | `P: Firmicutes`~Treatment.group + (1 \| Sampling.date) | ran_pars | Sampling.date | 8152.637 | *NA* | *NA* | *NA* | *NA* | *NA* |
| sd__Observation | P: Firmicutes | Phylum | `P: Firmicutes`~Treatment.group + (1 \| Sampling.date) | ran_pars | Residual | 13751.96 | *NA* | *NA* | *NA* | *NA* | *NA* |
| Treatment.groupCpG-methylated | P: Bacteroidota | Phylum | `P: Bacteroidota`~Treatment.group + (1 \| Sampling.date) | fixed | *NA* | -3076.235 | 3163.549 | -0.972 | 79.105 | 0.334 | 0.668 |
| Treatment.groupHypotonic Lysis | P: Bacteroidota | Phylum | `P: Bacteroidota`~Treatment.group + (1 \| Sampling.date) | fixed | *NA* | 371.856 | 3275.98 | 0.114 | 79.165 | 0.91 | 0.92 |
| Treatment.groupMolysis | P: Bacteroidota | Phylum | `P: Bacteroidota`~Treatment.group + (1 \| Sampling.date) | fixed | *NA* | -7096.056 | 3330.077 | -2.131 | 79.119 | 0.036 | 0.328 |
| Treatment.groupPBS | P: Bacteroidota | Phylum | `P: Bacteroidota`~Treatment.group + (1 \| Sampling.date) | fixed | *NA* | 2593.531 | 4174.859 | 0.621 | 80.013 | 0.536 | 0.772 |
| Treatment.groupZymo | P: Bacteroidota | Phylum | `P: Bacteroidota`~Treatment.group + (1 \| Sampling.date) | fixed | *NA* | 322.564 | 3218.48 | 0.1 | 79.156 | 0.92 | 0.92 |
| sd__(Intercept) | P: Bacteroidota | Phylum | `P: Bacteroidota`~Treatment.group + (1 \| Sampling.date) | ran_pars | Sampling.date | 6791.166 | *NA* | *NA* | *NA* | *NA* | *NA* |
| sd__Observation | P: Bacteroidota | Phylum | `P: Bacteroidota`~Treatment.group + (1 \| Sampling.date) | ran_pars | Residual | 9223.25 | *NA* | *NA* | *NA* | *NA* | *NA* |
| Treatment.groupCpG-methylated | P: Verrucomicrobiota | Phylum | `P: Verrucomicrobiota`~Treatment.group + (1 \| Sampling.date) | fixed | *NA* | -331.824 | 328.932 | -1.009 | 79.101 | 0.316 | 0.668 |
| Treatment.groupHypotonic Lysis | P: Verrucomicrobiota | Phylum | `P: Verrucomicrobiota`~Treatment.group + (1 \| Sampling.date) | fixed | *NA* | 155.919 | 340.613 | 0.458 | 79.169 | 0.648 | 0.812 |
| Treatment.groupMolysis | P: Verrucomicrobiota | Phylum | `P: Verrucomicrobiota`~Treatment.group + (1 \| Sampling.date) | fixed | *NA* | -123.525 | 346.244 | -0.357 | 79.117 | 0.722 | 0.85 |
| Treatment.groupPBS | P: Verrucomicrobiota | Phylum | `P: Verrucomicrobiota`~Treatment.group + (1 \| Sampling.date) | fixed | *NA* | 617.511 | 433.882 | 1.423 | 80.114 | 0.159 | 0.529 |
| Treatment.groupZymo | P: Verrucomicrobiota | Phylum | `P: Verrucomicrobiota`~Treatment.group + (1 \| Sampling.date) | fixed | *NA* | 430.479 | 334.636 | 1.286 | 79.158 | 0.202 | 0.577 |
| sd__(Intercept) | P: Verrucomicrobiota | Phylum | `P: Verrucomicrobiota`~Treatment.group + (1 \| Sampling.date) | ran_pars | Sampling.date | 659.593 | *NA* | *NA* | *NA* | *NA* | *NA* |
| sd__Observation | P: Verrucomicrobiota | Phylum | `P: Verrucomicrobiota`~Treatment.group + (1 \| Sampling.date) | ran_pars | Residual | 958.992 | *NA* | *NA* | *NA* | *NA* | *NA* |
| Treatment.groupCpG-methylated | C: Gammaproteobacteria | Class | `C: Gammaproteobacteria`~Treatment.group + (1 \| Sampling.date) | fixed | *NA* | 2128.412 | 10429.904 | 0.204 | 79.283 | 0.839 | 0.92 |
| Treatment.groupHypotonic Lysis | C: Gammaproteobacteria | Class | `C: Gammaproteobacteria`~Treatment.group + (1 \| Sampling.date) | fixed | *NA* | 3478.833 | 10785.511 | 0.323 | 79.713 | 0.748 | 0.897 |
| Treatment.groupMolysis | C: Gammaproteobacteria | Class | `C: Gammaproteobacteria`~Treatment.group + (1 \| Sampling.date) | fixed | *NA* | -16767.596 | 10976.09 | -1.528 | 79.349 | 0.131 | 0.392 |
| Treatment.groupPBS | C: Gammaproteobacteria | Class | `C: Gammaproteobacteria`~Treatment.group + (1 \| Sampling.date) | fixed | *NA* | 8040.849 | 13402.141 | 0.6 | 75.583 | 0.55 | 0.786 |
| Treatment.groupZymo | C: Gammaproteobacteria | Class | `C: Gammaproteobacteria`~Treatment.group + (1 \| Sampling.date) | fixed | *NA* | 19933.725 | 10599.829 | 1.881 | 79.578 | 0.064 | 0.239 |
| sd__(Intercept) | C: Gammaproteobacteria | Class | `C: Gammaproteobacteria`~Treatment.group + (1 \| Sampling.date) | ran_pars | Sampling.date | 5259.17 | *NA* | *NA* | *NA* | *NA* | *NA* |
| sd__Observation | C: Gammaproteobacteria | Class | `C: Gammaproteobacteria`~Treatment.group + (1 \| Sampling.date) | ran_pars | Residual | 30408.135 | *NA* | *NA* | *NA* | *NA* | *NA* |
| Treatment.groupCpG-methylated | C: Clostridia | Class | `C: Clostridia`~Treatment.group + (1 \| Sampling.date) | fixed | *NA* | 3434.471 | 4141.926 | 0.829 | 79.12 | 0.409 | 0.682 |
| Treatment.groupHypotonic Lysis | C: Clostridia | Class | `C: Clostridia`~Treatment.group + (1 \| Sampling.date) | fixed | *NA* | -4474.625 | 4288.376 | -1.043 | 79.23 | 0.3 | 0.626 |
| Treatment.groupMolysis | C: Clostridia | Class | `C: Clostridia`~Treatment.group + (1 \| Sampling.date) | fixed | *NA* | -679.848 | 4359.785 | -0.156 | 79.144 | 0.876 | 0.92 |
| Treatment.groupPBS | C: Clostridia | Class | `C: Clostridia`~Treatment.group + (1 \| Sampling.date) | fixed | *NA* | -1490.691 | 5449.07 | -0.274 | 80.603 | 0.785 | 0.906 |
| Treatment.groupZymo | C: Clostridia | Class | `C: Clostridia`~Treatment.group + (1 \| Sampling.date) | fixed | *NA* | -3002.219 | 4213.229 | -0.713 | 79.211 | 0.478 | 0.755 |
| sd__(Intercept) | C: Clostridia | Class | `C: Clostridia`~Treatment.group + (1 \| Sampling.date) | ran_pars | Sampling.date | 6235.061 | *NA* | *NA* | *NA* | *NA* | *NA* |
| sd__Observation | C: Clostridia | Class | `C: Clostridia`~Treatment.group + (1 \| Sampling.date) | ran_pars | Residual | 12075.686 | *NA* | *NA* | *NA* | *NA* | *NA* |
| Treatment.groupCpG-methylated | C: Alphaproteobacteria | Class | `C: Alphaproteobacteria`~Treatment.group + (1 \| Sampling.date) | fixed | *NA* | -914.059 | 1864.892 | -0.49 | 79.661 | 0.625 | 0.846 |
| Treatment.groupHypotonic Lysis | C: Alphaproteobacteria | Class | `C: Alphaproteobacteria`~Treatment.group + (1 \| Sampling.date) | fixed | *NA* | 1731.47 | 1927.498 | 0.898 | 80.107 | 0.372 | 0.656 |
| Treatment.groupMolysis | C: Alphaproteobacteria | Class | `C: Alphaproteobacteria`~Treatment.group + (1 \| Sampling.date) | fixed | *NA* | 7434.988 | 1962.418 | 3.789 | 79.721 | 0 | 0.009 |
| Treatment.groupPBS | C: Alphaproteobacteria | Class | `C: Alphaproteobacteria`~Treatment.group + (1 \| Sampling.date) | fixed | *NA* | 2398.978 | 2370.725 | 1.012 | 70.183 | 0.315 | 0.626 |
| Treatment.groupZymo | C: Alphaproteobacteria | Class | `C: Alphaproteobacteria`~Treatment.group + (1 \| Sampling.date) | fixed | *NA* | 2926.159 | 1894.653 | 1.544 | 79.949 | 0.126 | 0.392 |
| sd__(Intercept) | C: Alphaproteobacteria | Class | `C: Alphaproteobacteria`~Treatment.group + (1 \| Sampling.date) | ran_pars | Sampling.date | 626.238 | *NA* | *NA* | *NA* | *NA* | *NA* |
| sd__Observation | C: Alphaproteobacteria | Class | `C: Alphaproteobacteria`~Treatment.group + (1 \| Sampling.date) | ran_pars | Residual | 5437.049 | *NA* | *NA* | *NA* | *NA* | *NA* |
| Treatment.groupCpG-methylated | C: Bacteroidia | Class | `C: Bacteroidia`~Treatment.group + (1 \| Sampling.date) | fixed | *NA* | -3076.235 | 3163.549 | -0.972 | 79.105 | 0.334 | 0.626 |
| Treatment.groupHypotonic Lysis | C: Bacteroidia | Class | `C: Bacteroidia`~Treatment.group + (1 \| Sampling.date) | fixed | *NA* | 371.856 | 3275.98 | 0.114 | 79.165 | 0.91 | 0.92 |
| Treatment.groupMolysis | C: Bacteroidia | Class | `C: Bacteroidia`~Treatment.group + (1 \| Sampling.date) | fixed | *NA* | -7096.056 | 3330.077 | -2.131 | 79.119 | 0.036 | 0.217 |
| Treatment.groupPBS | C: Bacteroidia | Class | `C: Bacteroidia`~Treatment.group + (1 \| Sampling.date) | fixed | *NA* | 2593.531 | 4174.859 | 0.621 | 80.013 | 0.536 | 0.786 |
| Treatment.groupZymo | C: Bacteroidia | Class | `C: Bacteroidia`~Treatment.group + (1 \| Sampling.date) | fixed | *NA* | 322.564 | 3218.48 | 0.1 | 79.156 | 0.92 | 0.92 |
| sd__(Intercept) | C: Bacteroidia | Class | `C: Bacteroidia`~Treatment.group + (1 \| Sampling.date) | ran_pars | Sampling.date | 6791.166 | *NA* | *NA* | *NA* | *NA* | *NA* |
| sd__Observation | C: Bacteroidia | Class | `C: Bacteroidia`~Treatment.group + (1 \| Sampling.date) | ran_pars | Residual | 9223.25 | *NA* | *NA* | *NA* | *NA* | *NA* |
| Treatment.groupCpG-methylated | C: Verrucomicrobiae | Class | `C: Verrucomicrobiae`~Treatment.group + (1 \| Sampling.date) | fixed | *NA* | -331.824 | 328.932 | -1.009 | 79.101 | 0.316 | 0.626 |
| Treatment.groupHypotonic Lysis | C: Verrucomicrobiae | Class | `C: Verrucomicrobiae`~Treatment.group + (1 \| Sampling.date) | fixed | *NA* | 155.919 | 340.613 | 0.458 | 79.169 | 0.648 | 0.846 |
| Treatment.groupMolysis | C: Verrucomicrobiae | Class | `C: Verrucomicrobiae`~Treatment.group + (1 \| Sampling.date) | fixed | *NA* | -123.525 | 346.244 | -0.357 | 79.117 | 0.722 | 0.897 |
| Treatment.groupPBS | C: Verrucomicrobiae | Class | `C: Verrucomicrobiae`~Treatment.group + (1 \| Sampling.date) | fixed | *NA* | 617.511 | 433.882 | 1.423 | 80.114 | 0.159 | 0.432 |
| Treatment.groupZymo | C: Verrucomicrobiae | Class | `C: Verrucomicrobiae`~Treatment.group + (1 \| Sampling.date) | fixed | *NA* | 430.479 | 334.636 | 1.286 | 79.158 | 0.202 | 0.505 |
| sd__(Intercept) | C: Verrucomicrobiae | Class | `C: Verrucomicrobiae`~Treatment.group + (1 \| Sampling.date) | ran_pars | Sampling.date | 659.593 | *NA* | *NA* | *NA* | *NA* | *NA* |
| sd__Observation | C: Verrucomicrobiae | Class | `C: Verrucomicrobiae`~Treatment.group + (1 \| Sampling.date) | ran_pars | Residual | 958.992 | *NA* | *NA* | *NA* | *NA* | *NA* |
| Treatment.groupCpG-methylated | C: Bacilli | Class | `C: Bacilli`~Treatment.group + (1 \| Sampling.date) | fixed | *NA* | -6332.824 | 1997.846 | -3.17 | 79.258 | 0.002 | 0.019 |
| Treatment.groupHypotonic Lysis | C: Bacilli | Class | `C: Bacilli`~Treatment.group + (1 \| Sampling.date) | fixed | *NA* | -4097.261 | 2067.3 | -1.982 | 79.515 | 0.051 | 0.218 |
| Treatment.groupMolysis | C: Bacilli | Class | `C: Bacilli`~Treatment.group + (1 \| Sampling.date) | fixed | *NA* | -6569.648 | 2102.69 | -3.124 | 79.306 | 0.002 | 0.019 |
| Treatment.groupPBS | C: Bacilli | Class | `C: Bacilli`~Treatment.group + (1 \| Sampling.date) | fixed | *NA* | -5227.581 | 2600.549 | -2.01 | 80.544 | 0.048 | 0.218 |
| Treatment.groupZymo | C: Bacilli | Class | `C: Bacilli`~Treatment.group + (1 \| Sampling.date) | fixed | *NA* | -6594.561 | 2031.326 | -3.246 | 79.452 | 0.002 | 0.019 |
| sd__(Intercept) | C: Bacilli | Class | `C: Bacilli`~Treatment.group + (1 \| Sampling.date) | ran_pars | Sampling.date | 1633.343 | *NA* | *NA* | *NA* | *NA* | *NA* |
| sd__Observation | C: Bacilli | Class | `C: Bacilli`~Treatment.group + (1 \| Sampling.date) | ran_pars | Residual | 5824.672 | *NA* | *NA* | *NA* | *NA* | *NA* |
| Treatment.groupCpG-methylated | O: Enterobacterales | Order | `O: Enterobacterales`~Treatment.group + (1 \| Sampling.date) | fixed | *NA* | 6235.235 | 6222.28 | 1.002 | 79.061 | 0.319 | 0.702 |
| Treatment.groupHypotonic Lysis | O: Enterobacterales | Order | `O: Enterobacterales`~Treatment.group + (1 \| Sampling.date) | fixed | *NA* | -1426.302 | 6443.822 | -0.221 | 79.102 | 0.825 | 0.9 |
| Treatment.groupMolysis | O: Enterobacterales | Order | `O: Enterobacterales`~Treatment.group + (1 \| Sampling.date) | fixed | *NA* | -10015.397 | 6549.914 | -1.529 | 79.071 | 0.13 | 0.702 |
| Treatment.groupPBS | O: Enterobacterales | Order | `O: Enterobacterales`~Treatment.group + (1 \| Sampling.date) | fixed | *NA* | 7827.581 | 8220.349 | 0.952 | 79.723 | 0.344 | 0.702 |
| Treatment.groupZymo | O: Enterobacterales | Order | `O: Enterobacterales`~Treatment.group + (1 \| Sampling.date) | fixed | *NA* | 9996.46 | 6330.662 | 1.579 | 79.097 | 0.118 | 0.702 |
| sd__(Intercept) | O: Enterobacterales | Order | `O: Enterobacterales`~Treatment.group + (1 \| Sampling.date) | ran_pars | Sampling.date | 16350.068 | *NA* | *NA* | *NA* | *NA* | *NA* |
| sd__Observation | O: Enterobacterales | Order | `O: Enterobacterales`~Treatment.group + (1 \| Sampling.date) | ran_pars | Residual | 18140.909 | *NA* | *NA* | *NA* | *NA* | *NA* |
| Treatment.groupCpG-methylated | O: Burkholderiales | Order | `O: Burkholderiales`~Treatment.group + (1 \| Sampling.date) | fixed | *NA* | -3190.765 | 8155.103 | -0.391 | 79.434 | 0.697 | 0.85 |
| Treatment.groupHypotonic Lysis | O: Burkholderiales | Order | `O: Burkholderiales`~Treatment.group + (1 \| Sampling.date) | fixed | *NA* | 3554.728 | 8437.219 | 0.421 | 79.707 | 0.675 | 0.85 |
| Treatment.groupMolysis | O: Burkholderiales | Order | `O: Burkholderiales`~Treatment.group + (1 \| Sampling.date) | fixed | *NA* | -5840.027 | 8582.82 | -0.68 | 79.482 | 0.498 | 0.787 |
| Treatment.groupPBS | O: Burkholderiales | Order | `O: Burkholderiales`~Treatment.group + (1 \| Sampling.date) | fixed | *NA* | 759.122 | 10581.341 | 0.072 | 79.944 | 0.943 | 0.959 |
| Treatment.groupZymo | O: Burkholderiales | Order | `O: Burkholderiales`~Treatment.group + (1 \| Sampling.date) | fixed | *NA* | 7588.273 | 8290.758 | 0.915 | 79.634 | 0.363 | 0.702 |
| sd__(Intercept) | O: Burkholderiales | Order | `O: Burkholderiales`~Treatment.group + (1 \| Sampling.date) | ran_pars | Sampling.date | 5856.04 | *NA* | *NA* | *NA* | *NA* | *NA* |
| sd__Observation | O: Burkholderiales | Order | `O: Burkholderiales`~Treatment.group + (1 \| Sampling.date) | ran_pars | Residual | 23776.007 | *NA* | *NA* | *NA* | *NA* | *NA* |
| Treatment.groupCpG-methylated | O: Clostridiales | Order | `O: Clostridiales`~Treatment.group + (1 \| Sampling.date) | fixed | *NA* | 3434.471 | 4141.926 | 0.829 | 79.12 | 0.409 | 0.723 |
| Treatment.groupHypotonic Lysis | O: Clostridiales | Order | `O: Clostridiales`~Treatment.group + (1 \| Sampling.date) | fixed | *NA* | -4474.625 | 4288.376 | -1.043 | 79.23 | 0.3 | 0.702 |
| Treatment.groupMolysis | O: Clostridiales | Order | `O: Clostridiales`~Treatment.group + (1 \| Sampling.date) | fixed | *NA* | -679.848 | 4359.785 | -0.156 | 79.144 | 0.876 | 0.923 |
| Treatment.groupPBS | O: Clostridiales | Order | `O: Clostridiales`~Treatment.group + (1 \| Sampling.date) | fixed | *NA* | -1490.691 | 5449.07 | -0.274 | 80.603 | 0.785 | 0.872 |
| Treatment.groupZymo | O: Clostridiales | Order | `O: Clostridiales`~Treatment.group + (1 \| Sampling.date) | fixed | *NA* | -3002.219 | 4213.229 | -0.713 | 79.211 | 0.478 | 0.775 |
| sd__(Intercept) | O: Clostridiales | Order | `O: Clostridiales`~Treatment.group + (1 \| Sampling.date) | ran_pars | Sampling.date | 6235.061 | *NA* | *NA* | *NA* | *NA* | *NA* |
| sd__Observation | O: Clostridiales | Order | `O: Clostridiales`~Treatment.group + (1 \| Sampling.date) | ran_pars | Residual | 12075.686 | *NA* | *NA* | *NA* | *NA* | *NA* |
| Treatment.groupCpG-methylated | O: Rhizobiales | Order | `O: Rhizobiales`~Treatment.group + (1 \| Sampling.date) | fixed | *NA* | -282.294 | 578.508 | -0.488 | 79.196 | 0.627 | 0.85 |
| Treatment.groupHypotonic Lysis | O: Rhizobiales | Order | `O: Rhizobiales`~Treatment.group + (1 \| Sampling.date) | fixed | *NA* | 368.304 | 598.345 | 0.616 | 79.593 | 0.54 | 0.831 |
| Treatment.groupMolysis | O: Rhizobiales | Order | `O: Rhizobiales`~Treatment.group + (1 \| Sampling.date) | fixed | *NA* | 3577.617 | 608.82 | 5.876 | 79.261 | 0 | <0.001 |
| Treatment.groupPBS | O: Rhizobiales | Order | `O: Rhizobiales`~Treatment.group + (1 \| Sampling.date) | fixed | *NA* | 571.677 | 746.244 | 0.766 | 77.491 | 0.446 | 0.743 |
| Treatment.groupZymo | O: Rhizobiales | Order | `O: Rhizobiales`~Treatment.group + (1 \| Sampling.date) | fixed | *NA* | 867.405 | 588.008 | 1.475 | 79.476 | 0.144 | 0.702 |
| sd__(Intercept) | O: Rhizobiales | Order | `O: Rhizobiales`~Treatment.group + (1 \| Sampling.date) | ran_pars | Sampling.date | 334.612 | *NA* | *NA* | *NA* | *NA* | *NA* |
| sd__Observation | O: Rhizobiales | Order | `O: Rhizobiales`~Treatment.group + (1 \| Sampling.date) | ran_pars | Residual | 1686.625 | *NA* | *NA* | *NA* | *NA* | *NA* |
| Treatment.groupCpG-methylated | O: Rhodobacterales | Order | `O: Rhodobacterales`~Treatment.group + (1 \| Sampling.date) | fixed | *NA* | -397.647 | 505.816 | -0.786 | 79.05 | 0.434 | 0.743 |
| Treatment.groupHypotonic Lysis | O: Rhodobacterales | Order | `O: Rhodobacterales`~Treatment.group + (1 \| Sampling.date) | fixed | *NA* | -270.907 | 523.779 | -0.517 | 79.119 | 0.606 | 0.85 |
| Treatment.groupMolysis | O: Rhodobacterales | Order | `O: Rhodobacterales`~Treatment.group + (1 \| Sampling.date) | fixed | *NA* | 176.036 | 532.439 | 0.331 | 79.066 | 0.742 | 0.856 |
| Treatment.groupPBS | O: Rhodobacterales | Order | `O: Rhodobacterales`~Treatment.group + (1 \| Sampling.date) | fixed | *NA* | -254.37 | 667.208 | -0.381 | 80.088 | 0.704 | 0.85 |
| Treatment.groupZymo | O: Rhodobacterales | Order | `O: Rhodobacterales`~Treatment.group + (1 \| Sampling.date) | fixed | *NA* | 712.266 | 514.588 | 1.384 | 79.108 | 0.17 | 0.702 |
| sd__(Intercept) | O: Rhodobacterales | Order | `O: Rhodobacterales`~Treatment.group + (1 \| Sampling.date) | ran_pars | Sampling.date | 1015.134 | *NA* | *NA* | *NA* | *NA* | *NA* |
| sd__Observation | O: Rhodobacterales | Order | `O: Rhodobacterales`~Treatment.group + (1 \| Sampling.date) | ran_pars | Residual | 1474.695 | *NA* | *NA* | *NA* | *NA* | *NA* |
| Treatment.groupCpG-methylated | O: Sphingomonadales | Order | `O: Sphingomonadales`~Treatment.group + (1 \| Sampling.date) | fixed | *NA* | -234.118 | 1464.869 | -0.16 | 81 | 0.873 | 0.923 |
| Treatment.groupHypotonic Lysis | O: Sphingomonadales | Order | `O: Sphingomonadales`~Treatment.group + (1 \| Sampling.date) | fixed | *NA* | 1561.808 | 1512.91 | 1.032 | 81 | 0.305 | 0.702 |
| Treatment.groupMolysis | O: Sphingomonadales | Order | `O: Sphingomonadales`~Treatment.group + (1 \| Sampling.date) | fixed | *NA* | 3737.37 | 1541.348 | 2.425 | 81 | 0.018 | 0.211 |
| Treatment.groupPBS | O: Sphingomonadales | Order | `O: Sphingomonadales`~Treatment.group + (1 \| Sampling.date) | fixed | *NA* | 1544.066 | 1831.086 | 0.843 | 81 | 0.402 | 0.723 |
| Treatment.groupZymo | O: Sphingomonadales | Order | `O: Sphingomonadales`~Treatment.group + (1 \| Sampling.date) | fixed | *NA* | 1328.316 | 1487.581 | 0.893 | 81 | 0.375 | 0.702 |
| sd__(Intercept) | O: Sphingomonadales | Order | `O: Sphingomonadales`~Treatment.group + (1 \| Sampling.date) | ran_pars | Sampling.date | 0 | *NA* | *NA* | *NA* | *NA* | *NA* |
| sd__Observation | O: Sphingomonadales | Order | `O: Sphingomonadales`~Treatment.group + (1 \| Sampling.date) | ran_pars | Residual | 4270.79 | *NA* | *NA* | *NA* | *NA* | *NA* |
| Treatment.groupCpG-methylated | O: Pseudomonadales | Order | `O: Pseudomonadales`~Treatment.group + (1 \| Sampling.date) | fixed | *NA* | -696.882 | 1757.36 | -0.397 | 79.084 | 0.693 | 0.85 |
| Treatment.groupHypotonic Lysis | O: Pseudomonadales | Order | `O: Pseudomonadales`~Treatment.group + (1 \| Sampling.date) | fixed | *NA* | 2287.735 | 1819.611 | 1.257 | 79.177 | 0.212 | 0.702 |
| Treatment.groupMolysis | O: Pseudomonadales | Order | `O: Pseudomonadales`~Treatment.group + (1 \| Sampling.date) | fixed | *NA* | -1737.954 | 1849.82 | -0.94 | 79.105 | 0.35 | 0.702 |
| Treatment.groupPBS | O: Pseudomonadales | Order | `O: Pseudomonadales`~Treatment.group + (1 \| Sampling.date) | fixed | *NA* | 3372.543 | 2314.571 | 1.457 | 80.408 | 0.149 | 0.702 |
| Treatment.groupZymo | O: Pseudomonadales | Order | `O: Pseudomonadales`~Treatment.group + (1 \| Sampling.date) | fixed | *NA* | 2369.827 | 1787.706 | 1.326 | 79.162 | 0.189 | 0.702 |
| sd__(Intercept) | O: Pseudomonadales | Order | `O: Pseudomonadales`~Treatment.group + (1 \| Sampling.date) | ran_pars | Sampling.date | 2943.947 | *NA* | *NA* | *NA* | *NA* | *NA* |
| sd__Observation | O: Pseudomonadales | Order | `O: Pseudomonadales`~Treatment.group + (1 \| Sampling.date) | ran_pars | Residual | 5123.539 | *NA* | *NA* | *NA* | *NA* | *NA* |
| Treatment.groupCpG-methylated | O: Xanthomonadales | Order | `O: Xanthomonadales`~Treatment.group + (1 \| Sampling.date) | fixed | *NA* | -219.176 | 381.955 | -0.574 | 79.121 | 0.568 | 0.85 |
| Treatment.groupHypotonic Lysis | O: Xanthomonadales | Order | `O: Xanthomonadales`~Treatment.group + (1 \| Sampling.date) | fixed | *NA* | -403.965 | 395.485 | -1.021 | 79.213 | 0.31 | 0.702 |
| Treatment.groupMolysis | O: Xanthomonadales | Order | `O: Xanthomonadales`~Treatment.group + (1 \| Sampling.date) | fixed | *NA* | 463.454 | 402.05 | 1.153 | 79.141 | 0.252 | 0.702 |
| Treatment.groupPBS | O: Xanthomonadales | Order | `O: Xanthomonadales`~Treatment.group + (1 \| Sampling.date) | fixed | *NA* | -60.911 | 503.056 | -0.121 | 80.421 | 0.904 | 0.935 |
| Treatment.groupZymo | O: Xanthomonadales | Order | `O: Xanthomonadales`~Treatment.group + (1 \| Sampling.date) | fixed | *NA* | 144.315 | 388.55 | 0.371 | 79.197 | 0.711 | 0.85 |
| sd__(Intercept) | O: Xanthomonadales | Order | `O: Xanthomonadales`~Treatment.group + (1 \| Sampling.date) | ran_pars | Sampling.date | 639.098 | *NA* | *NA* | *NA* | *NA* | *NA* |
| sd__Observation | O: Xanthomonadales | Order | `O: Xanthomonadales`~Treatment.group + (1 \| Sampling.date) | ran_pars | Residual | 1113.58 | *NA* | *NA* | *NA* | *NA* | *NA* |
| Treatment.groupCpG-methylated | O: Flavobacteriales | Order | `O: Flavobacteriales`~Treatment.group + (1 \| Sampling.date) | fixed | *NA* | -2223.529 | 2476.182 | -0.898 | 79.1 | 0.372 | 0.702 |
| Treatment.groupHypotonic Lysis | O: Flavobacteriales | Order | `O: Flavobacteriales`~Treatment.group + (1 \| Sampling.date) | fixed | *NA* | 114.525 | 2564.144 | 0.045 | 79.164 | 0.964 | 0.964 |
| Treatment.groupMolysis | O: Flavobacteriales | Order | `O: Flavobacteriales`~Treatment.group + (1 \| Sampling.date) | fixed | *NA* | -6078.858 | 2606.518 | -2.332 | 79.115 | 0.022 | 0.222 |
| Treatment.groupPBS | O: Flavobacteriales | Order | `O: Flavobacteriales`~Treatment.group + (1 \| Sampling.date) | fixed | *NA* | 1268.234 | 3266.853 | 0.388 | 80.072 | 0.699 | 0.85 |
| Treatment.groupZymo | O: Flavobacteriales | Order | `O: Flavobacteriales`~Treatment.group + (1 \| Sampling.date) | fixed | *NA* | 1095.513 | 2519.145 | 0.435 | 79.154 | 0.665 | 0.85 |
| sd__(Intercept) | O: Flavobacteriales | Order | `O: Flavobacteriales`~Treatment.group + (1 \| Sampling.date) | ran_pars | Sampling.date | 5098.423 | *NA* | *NA* | *NA* | *NA* | *NA* |
| sd__Observation | O: Flavobacteriales | Order | `O: Flavobacteriales`~Treatment.group + (1 \| Sampling.date) | ran_pars | Residual | 7219.25 | *NA* | *NA* | *NA* | *NA* | *NA* |
| Treatment.groupCpG-methylated | O: Cytophagales | Order | `O: Cytophagales`~Treatment.group + (1 \| Sampling.date) | fixed | *NA* | -852.706 | 844.672 | -1.01 | 79.105 | 0.316 | 0.702 |
| Treatment.groupHypotonic Lysis | O: Cytophagales | Order | `O: Cytophagales`~Treatment.group + (1 \| Sampling.date) | fixed | *NA* | 262.069 | 874.694 | 0.3 | 79.164 | 0.765 | 0.866 |
| Treatment.groupMolysis | O: Cytophagales | Order | `O: Cytophagales`~Treatment.group + (1 \| Sampling.date) | fixed | *NA* | -1016.924 | 889.136 | -1.144 | 79.119 | 0.256 | 0.702 |
| Treatment.groupPBS | O: Cytophagales | Order | `O: Cytophagales`~Treatment.group + (1 \| Sampling.date) | fixed | *NA* | 1352.04 | 1114.759 | 1.213 | 80 | 0.229 | 0.702 |
| Treatment.groupZymo | O: Cytophagales | Order | `O: Cytophagales`~Treatment.group + (1 \| Sampling.date) | fixed | *NA* | -769.366 | 859.341 | -0.895 | 79.155 | 0.373 | 0.702 |
| sd__(Intercept) | O: Cytophagales | Order | `O: Cytophagales`~Treatment.group + (1 \| Sampling.date) | ran_pars | Sampling.date | 1830.008 | *NA* | *NA* | *NA* | *NA* | *NA* |
| sd__Observation | O: Cytophagales | Order | `O: Cytophagales`~Treatment.group + (1 \| Sampling.date) | ran_pars | Residual | 2462.621 | *NA* | *NA* | *NA* | *NA* | *NA* |
| Treatment.groupCpG-methylated | O: Verrucomicrobiales | Order | `O: Verrucomicrobiales`~Treatment.group + (1 \| Sampling.date) | fixed | *NA* | -331.824 | 328.932 | -1.009 | 79.101 | 0.316 | 0.702 |
| Treatment.groupHypotonic Lysis | O: Verrucomicrobiales | Order | `O: Verrucomicrobiales`~Treatment.group + (1 \| Sampling.date) | fixed | *NA* | 155.919 | 340.613 | 0.458 | 79.169 | 0.648 | 0.85 |
| Treatment.groupMolysis | O: Verrucomicrobiales | Order | `O: Verrucomicrobiales`~Treatment.group + (1 \| Sampling.date) | fixed | *NA* | -123.525 | 346.244 | -0.357 | 79.117 | 0.722 | 0.85 |
| Treatment.groupPBS | O: Verrucomicrobiales | Order | `O: Verrucomicrobiales`~Treatment.group + (1 \| Sampling.date) | fixed | *NA* | 617.511 | 433.882 | 1.423 | 80.114 | 0.159 | 0.702 |
| Treatment.groupZymo | O: Verrucomicrobiales | Order | `O: Verrucomicrobiales`~Treatment.group + (1 \| Sampling.date) | fixed | *NA* | 430.479 | 334.636 | 1.286 | 79.158 | 0.202 | 0.702 |
| sd__(Intercept) | O: Verrucomicrobiales | Order | `O: Verrucomicrobiales`~Treatment.group + (1 \| Sampling.date) | ran_pars | Sampling.date | 659.593 | *NA* | *NA* | *NA* | *NA* | *NA* |
| sd__Observation | O: Verrucomicrobiales | Order | `O: Verrucomicrobiales`~Treatment.group + (1 \| Sampling.date) | ran_pars | Residual | 958.992 | *NA* | *NA* | *NA* | *NA* | *NA* |
| Treatment.groupCpG-methylated | O: Mycoplasmatales | Order | `O: Mycoplasmatales`~Treatment.group + (1 \| Sampling.date) | fixed | *NA* | -6332.824 | 1997.846 | -3.17 | 79.258 | 0.002 | 0.037 |
| Treatment.groupHypotonic Lysis | O: Mycoplasmatales | Order | `O: Mycoplasmatales`~Treatment.group + (1 \| Sampling.date) | fixed | *NA* | -4097.261 | 2067.3 | -1.982 | 79.515 | 0.051 | 0.382 |
| Treatment.groupMolysis | O: Mycoplasmatales | Order | `O: Mycoplasmatales`~Treatment.group + (1 \| Sampling.date) | fixed | *NA* | -6569.648 | 2102.69 | -3.124 | 79.306 | 0.002 | 0.037 |
| Treatment.groupPBS | O: Mycoplasmatales | Order | `O: Mycoplasmatales`~Treatment.group + (1 \| Sampling.date) | fixed | *NA* | -5227.581 | 2600.549 | -2.01 | 80.544 | 0.048 | 0.382 |
| Treatment.groupZymo | O: Mycoplasmatales | Order | `O: Mycoplasmatales`~Treatment.group + (1 \| Sampling.date) | fixed | *NA* | -6594.561 | 2031.326 | -3.246 | 79.452 | 0.002 | 0.037 |
| sd__(Intercept) | O: Mycoplasmatales | Order | `O: Mycoplasmatales`~Treatment.group + (1 \| Sampling.date) | ran_pars | Sampling.date | 1633.343 | *NA* | *NA* | *NA* | *NA* | *NA* |
| sd__Observation | O: Mycoplasmatales | Order | `O: Mycoplasmatales`~Treatment.group + (1 \| Sampling.date) | ran_pars | Residual | 5824.672 | *NA* | *NA* | *NA* | *NA* | *NA* |
